# Fourteen Years of R/qtl: Just Barely Sustainable

**DOI:** 10.5334/jors.at

**Published:** 2014-07-09

**Authors:** Karl W. Broman

**Affiliations:** * Department of Biostatistics and Medical Informatics, University of Wisconsin-Madison, United States, kbroman@biostat.wisc.edu

**Keywords:** QTL mapping, software, quantitative trait loci, sustainable software

## Abstract

R/qtl is an R package for mapping quantitative trait loci (genetic loci that contribute to variation in quantitative traits) in experimental crosses. Its development began in 2000. There have been 38 software releases since 2001. The latest release contains 35k lines of R code and 24k lines of C code, plus 15k lines of code for the documentation. Challenges in the development and maintenance of the software are discussed. A key to the success of R/qtl is that it remains a central tool for the chief developer's own research work, and so its maintenance is of selfish importance.

## Introduction

If two inbred strains (for example, of plants) show consistent differences in a quantitative trait, one can be confident that the difference is genetic. An experimental cross between the two strains can be used to identify the genetic loci (called quantitative trait loci, QTL) that contribute to the trait difference: we seek genomic regions for which genotypes are associated with the trait.

**Figure 1:**
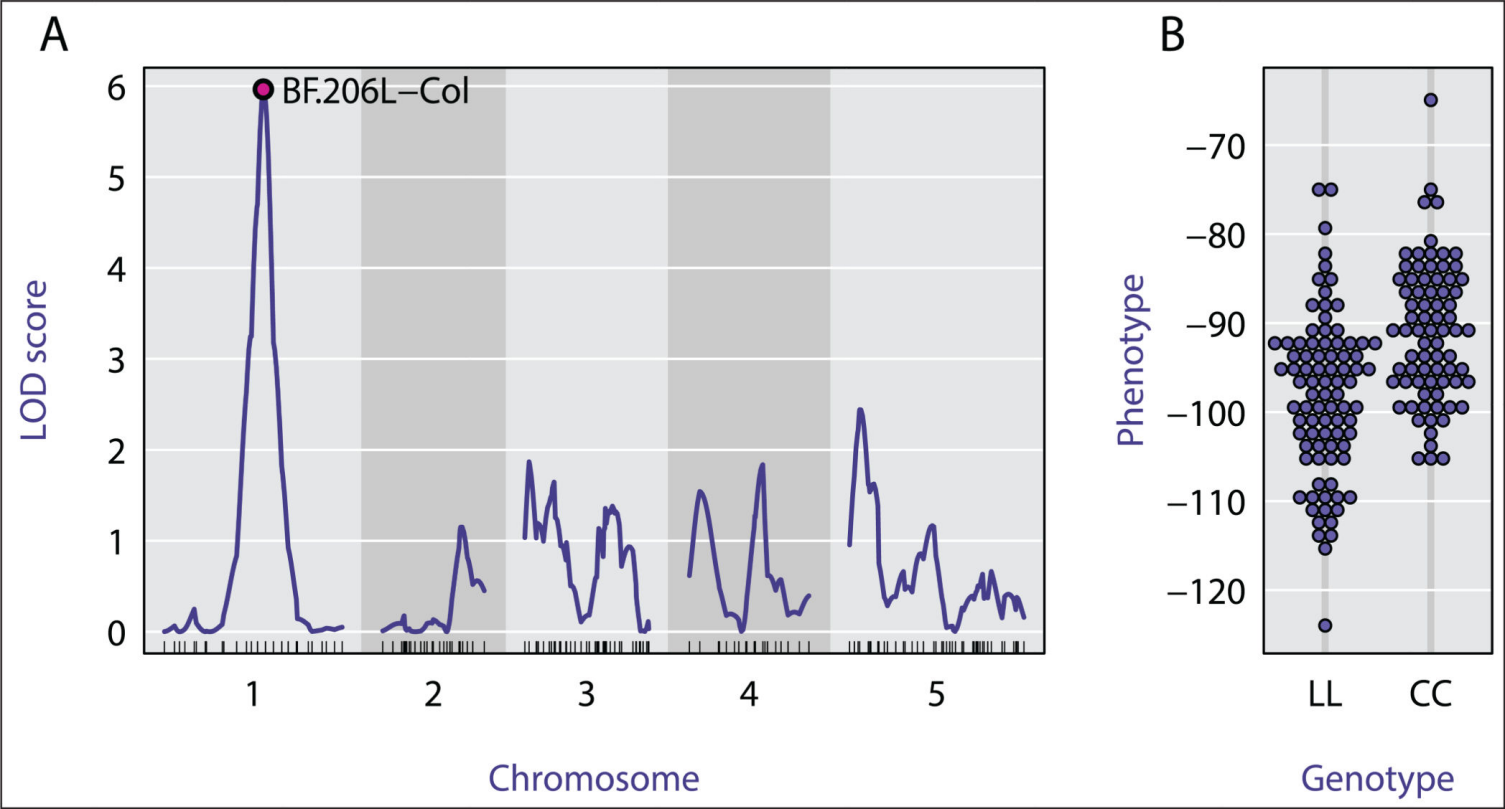
Typical analysis results from R/qtl. A: LOD curves across the genome, measuring association between phenotype and genotype across, and B: Association between genotype and phenotype at the marker with the strongest association. The data are from [1]; panel B was created using the R package beeswarm.

As an illustration, Figure 1 displays the results of QTL analysis with data from [1], on gravit-ropism in Arabidopsis. LOD curves measuring the strength of association between phenotype and genotype are displayed in Figure 1A; a plot of phenotype vs. genotype at the marker exhibiting the strongest association is shown in Figure 1B.

Numerous software packages for QTL analysis are available, some commercial (e.g., MapQTL and MultiQTL) and some free (e.g. Mapmaker/QTL, and QTL Cartographer).

My own QTL mapping software, R/qtl [2], is developed as an add-on package to the widely used general statistical software, R [3]. The software is open source, licensed under GPL3, and currently hosted on GitHub.

## History

I became interested in QTL mapping in graduate school, twenty years ago. Mark Neff introduced me to Lander and Botstein’s paper on interval mapping [4], which remains the most commonly used QTL analysis method.

In the fall of 1999, I joined the Department of Biostatistics at Johns Hopkins University as an assistant professor. In February, 2000, Gary Churchill visited me from the Jackson Laboratory, and we discussed our shared interest in QTL analysis methods and the need for more advanced software. Gary suggested that we write our own QTL mapping package. He was thinking Matlab, but I was keen to use R. (R version 1.0 was released the following week.) R won out over Matlab largely because I developed a working prototype more quickly. I had recently written some C code implementing the hidden Markov model (HMM) technology [5] for the treatment of missing genotype information for QTL analysis in experimental crosses. This served as the starting point for the package.

Our main goal was for the software to enable the exploration of multiple-QTL models. We also wanted it to be easily extendible as new methods were developed. My initial concept was to implement all QTL mapping methods, good and bad, so that their performance could be compared within a single package.

Much of the development of R/qtl occurred during a three-month sabbatical I spent at the Jackson Laboratory in Fall, 2001. (It is easy to remember the year, because I was there on 11 September 2001.) Hao Wu, a software engineer working with Gary from 2001–2005, contributed a great deal to the core of R/qtl. In 2009–2010, Danny Arends (with some assistance from Pjotr Prins and me) incorporated Ritsert Jansen’s MQM code [6, 7, 8], previously available only in commercial software, as part of R/qtl [9].

## About me

I am an applied statistician. My primary interest is in helping other scientists answer questions using data. But that generally requires the development of new statistical methods, and such methods must be implemented in software. Thus, I spend a considerable amount of time programming.

I have little formal training in programming or software engineering, and I am not a specialist in computational statistics. But I think it’s important for an applied statistician to be self-sufficient: We can’t rely on others to develop the tools we need but must be able to do that ourselves.

## Strengths

R/qtl has a number of strengths. It is comprehensive: It includes implementations of many QTL mapping methods, and it has a number of tools for the fit and exploration of multiple-QTL models. It has extensive facilities for data diagnostics and visualizations. It can be extended, as the results of important intermediate calculations, that serve as the basis for any QTL mapping method, are exposed to the user.

**Figure 2:**
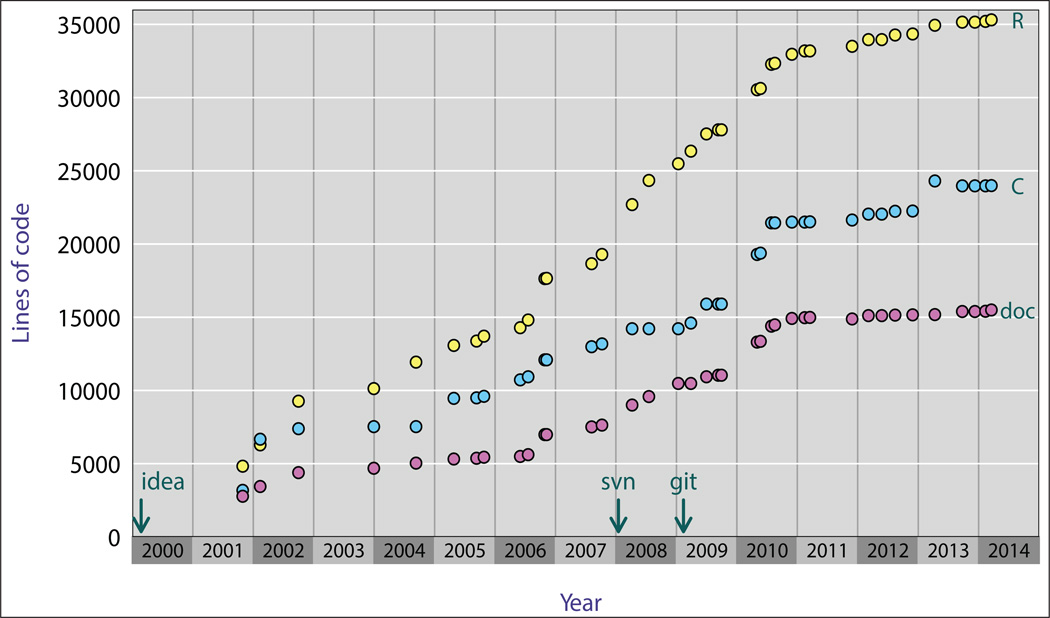
Numbers of lines of code in released versions of R/qtl over time. (Yellow and blue correspond to R and C code, respectively; pink is for the R documentation files.)

The central calculations are coded in C, for speed, but R is used for manipulating data objects and for graphics. Figure 2 displays the growth of code in R/qtl over time, as the number lines of code in R and C, as well as in the R documentation files. Ignoring the documentation files, the code is about 60% R and 40% C.

Developing the software as a package for R has a number of advantages for the developer, particularly to make use of R’s facilities for graphics and input/output, as well as its extensive math/stat software library. The R packaging system and the Comprehensive R Archive Network (CRAN, http://cran.r-project.org) simplifies the software installation process and makes it easy to provide documentation.

The user also benefits by having the QTL mapping software embedded within general statistical software: for further data analysis before and after the QTL analysis, and for developing specially-tailored visualizations. R also provides an excellent interactive environment for data analysis.

A number of related packages are coordinated with R/ qtl, using a common data structure or input file format and some shared functions. Examples include qtlbim [10], wgaim [11], and dlmap [12].

## Weaknesses

R/qtl also has a number of weaknesses. There has largely been one developer (who doubles as the support staff), and so many desired features have not been implemented. We never wrote formal specifications for the internal data formats, which makes it more difficult for others to contribute code to the project.

There have been a number of memory management issues, particularly regarding the copying of large datasets in memory as it is moved from R to C. This can reduce performance in extremely high-dimensional applications.

The biggest weakness is that the central data structure is too restrictive. This makes it difficult to extend the software beyond the simplest types of experimental crosses.

## Some really bad code

More embarrassing than the above weaknesses is that, while R/qtl contains some good code (like the HMM implementation), there is also some really terrible code. I’ve learned a great deal about programming while developing R/qtl, but it’s hard to go back and replace old code. (And some of this code was obviously bad when written and just should have been constructed with greater care.)

The worst piece of code (now fixed: see the commit in the git repository on GitHub, https://github.com/kbroman/qtl/commit/4cd486) involved a really stupid approach to find the first non-blank element in a vector of character strings. The code worked fine in small datasets, and so it took a while to discover the problem. (Open source means everyone can see my stupid mistakes. Version control means everyone can see every stupid mistake I’ve ever made.)

In many cases, functions aren’t split up into short reusable functions the way they should be, and there is a lot of repeated code. And some of the functions have very long lists of arguments, which can be really intimidating to users.

In lots of cases, bugs were fixed by adding little band-aids of code. Functions get longer and longer to handle more and more special cases, to the point that they are nearly unreadable. These very long, complex functions are a barrier to the further development of the software.

The worst offender is scantwo (), for performing a two-dimensional genome scan for pairs of QTL. The R function is 1402 lines long (that is 4% of the R code in the package). And the R code is just moving data and results around. The actual calculations are performed in C, in a series of files that comprise 4725 lines, which is 20% of the C code in the package.

## Version control

In the first eight years of developing R/qtl, I didn’t use formal version control. Initially, I was simply editing the code in place and saving copies of releases when I sent them to the R Archive. Incorporating others’ contributions was often a hassle.

In the fall of 2006, Śaunak Sen and I used a Subversion repository to facilitate the development of our book about QTL mapping and R/qtl [13], but it wasn’t until January, 2008, that I began to use subversion for R/qtl, as well. In February, 2009, Pjotr Prins helped me to switch to git and to place the R/qtl repository on GitHub.

I’m not sure how I managed the project before adopting version control. Version control makes it so easy to try things out without fear of breaking working code. And collaboration on software development is terribly tedious and inefficient without version control.

## User support

Helping users to solve their research problems can be quite rewarding. But it can also require considerable patience. I respond to several queries per week about R/ qtl, mainly through a Google Group that was started in September, 2004, but many others directly via email.

I had hoped that the R/qtl discussion group would become a network of folks answering each others’ questions about R/qtl and QTL mapping, but I am basically the only one answering questions. I think that’s partly because, to avoid spam, I moderate all messages, at which point I generally try to answer them. Thus, participants see my answer at basically the same time as they see the original question. But at least these electronic conversations are public and searchable.

I’ve helped many people revise their data files for use with R/qtl. It’s very hard to predict what might go wrong in data import and to provide appropriate error messages.

Questions are often frustrating. They may provide too little detail (“I tried such-and-such but *it didn’t work*.”) or too much (“Could you please look at the attached 25-page Word document containing code and output and tell me if I’m doing something wrong?”) Many questions are not so much about the software but are more general scientific or data analysis questions.

Many questions would be answered by a careful reading of the documentation, but software documentation is often dreadfully boring. I’ve learned that the most popular documentation are the short tutorials with practical examples clearly illustrating important tools. These are time-consuming to create (and maintain).

Some things are *possible* in R/qtl but are not well documented and not really feasible except for users with considerable programming and analysis experience. It feels wrong to say, “That is possible, but I don’t have the time to explain how.”

It was helpful to have written a book about QTL mapping and R/qtl [13], but it’s frustrating to watch the publisher nearly double its street price. If I could do it again, I would self-publish.

## Sustainable academic software

Why put so much effort into software development? The principal advantage is the software itself: QTL analysis is the focus of much of my own research, and software that is easy for others to use is also easy for me to use. Second, R/qtl provides a platform for me to distribute implementations of QTL mapping methods that I develop, such as the two-part model of [14] and the model selection methods of [15] and [16]. Third, the software has led to many interesting consulting-type interactions and a good number of collaborations (leading to co-authorship on papers and some grant support). Fourth, my own methods grant is much more attractive with a successful software aim. Finally, the software supports others’ research, and my primary duty as a statistician and an academic is to help others.

I think the key to the sustainability of piece of scientific software is that the developer continues to use the software for his/her own research. So often, the developer moves on to other research problems, and the software is orphaned.

But software development requires considerable time and support. Often, researchers don’t recognize the many indirect benefits of building useful software tools, nor the long-term benefits of devoting extra time to the construction of *good* software. The reviewers of grant proposals may recognize the value of software development, but generally it can not be the sole research effort, and funding agencies often over-emphasize innovation, which can make it difficult to obtain support for software maintenance. Moreover, academic culture encourages researchers to create independent software packages rather than contribute to existing ones.

The biggest open question, for me personally, is how to encourage the formation of a community around a given software development effort: for coding, for answering users’ questions, and for developing improved documentation and tutorials.

## Future

As Fred Brooks said in *The Mythical Man Month,* “Plan to throw one away; you will anyhow.” [17], Ch. 11) This was restated by Eric Raymond in *The Cathedral and The Bazaar.* “You often don’t really understand the problem until after the first time you implement a solution. The second time, maybe you know enough to do it right.” [18]

In collaboration with Danny Arends and Pjotr Prins, I’ve initiated a reimplementation of R/qtl, with a focus on high-dimensional data and more modern cross designs, such as the Collaborative Cross [19] and the Mouse Diversity Outbred Population [20]. We will also focus on interactive graphical tools, implemented with the Javascript library, D3.

There has been a renewal of interest in QTL analysis, particularly with the growth of eQTL analysis, in which genome-wide gene expression measures are treated as phenotypes (see, for example, [21, 22]). We hope that our new software will become a popular platform for the analysis of large-scale eQTL data, as R/qtl has been for more traditional-sized QTL projects.
